# CSEA-DB: an omnibus for human complex trait and cell type associations

**DOI:** 10.1093/nar/gkaa1064

**Published:** 2020-11-19

**Authors:** Yulin Dai, Ruifeng Hu, Astrid Marilyn Manuel, Andi Liu, Peilin Jia, Zhongming Zhao

**Affiliations:** Center for Precision Health, School of Biomedical Informatics, The University of Texas Health Science Center at Houston, Houston, TX 77030, USA; Center for Precision Health, School of Biomedical Informatics, The University of Texas Health Science Center at Houston, Houston, TX 77030, USA; Center for Precision Health, School of Biomedical Informatics, The University of Texas Health Science Center at Houston, Houston, TX 77030, USA; Human Genetics Center, School of Public Health, The University of Texas Health Science Center at Houston, Houston, TX 77030, USA; Center for Precision Health, School of Biomedical Informatics, The University of Texas Health Science Center at Houston, Houston, TX 77030, USA; Center for Precision Health, School of Biomedical Informatics, The University of Texas Health Science Center at Houston, Houston, TX 77030, USA; Human Genetics Center, School of Public Health, The University of Texas Health Science Center at Houston, Houston, TX 77030, USA; MD Anderson Cancer Center UTHealth Graduate School of Biomedical Sciences, Houston, TX 77030, USA

## Abstract

During the past decade, genome-wide association studies (GWAS) have identified many genetic variants with susceptibility to several thousands of complex diseases or traits. The genetic regulation of gene expression is highly tissue-specific and cell type-specific. Recently, single-cell technology has paved the way to dissect cellular heterogeneity in human tissues. Here, we present a reference database for GWAS trait-associated cell type-specificity, named Cell type-Specific Enrichment Analysis DataBase (CSEA-DB, available at https://bioinfo.uth.edu/CSEADB/). Specifically, we curated total of 5120 GWAS summary statistics data for a wide range of human traits and diseases followed by rigorous quality control. We further collected >900 000 cells from the leading consortia such as Human Cell Landscape, Human Cell Atlas, and extensive literature mining, including 752 tissue cell types from 71 adult and fetal tissues across 11 human organ systems. The tissues and cell types were annotated with Uberon and Cell Ontology. By applying our deTS algorithm, we conducted 10 250 480 times of trait-cell type associations, reporting a total of 598 (11.68%) GWAS traits with at least one significantly associated cell type. In summary, CSEA-DB could serve as a repository of association map for human complex traits and their underlying cell types, manually curated GWAS, and single-cell transcriptome resources.

## INTRODUCTION

The past decade has witnessed great success in genome-wide association studies (GWAS) which reported thousands of genetic variants with statistical association with a few thousands of human complex diseases and traits. However, where and how these genetic factors manifest their impacts on the molecular changes remain mostly elusive. Previous studies have discovered that genetic variants tend to regulate the gene expression or function in specific tissues and cell types (1,2). The accurate assessment of disease-associated tissues or cell types becomes a critical step to understanding the etiology of these human complex diseases and traits (3,4). Recently, we successfully developed a t-statistics-based method ‘decoding the tissue-specificity’ (deTS) to measure the tissue-specific enrichment of 26 human complex diseases utilizing GWAS summary statistics and tissue gene expression profiling (5). Later, we expanded this method to assess the tissue-specific enrichment of ∼5000 collected GWAS over ∼70 tissues curated from the Genotype-Tissue Expression project (GTEx) (2) and Encyclopedia of DNA Elements project (ENCODE) (6). All the trait and tissue associations are stored in our Tissue-Specific Enrichment Analysis DataBase (TSEA-DB, https://bioinfo.uth.edu/TSEADB/) (7). However, due to the heterogeneity within the tissue, the bulk RNA-seq of tissue might not fully reflect the underlying biological basis.

Human bodies are composed of 11 major organ systems, ∼100 organs/tissues, and more than 100 unique cell types or thousands of sub-cell types (8). In recent years, large-scale single-cell transcriptome data have been generated by several pioneer studies (9–11) and multiple international consortia (e.g. Human Cell Atlas, Human Lung Cell Atlas, and Human Cell Landscape) (12–14). These studies aim to characterize the molecular features of the cell types in human major tissues, which provides us rich resources to decode the cell type-specificity of human cell types and harness the genetic implications underlying human complex diseases and traits.

In this work, we aim to systematically explore the genetic signals of complex traits and diseases underlying human cell types. We conduct the following approaches. (i) We updated our GWAS summary statistics collection. (ii) We curated these large-scale single-cell transcriptome datasets of human tissues and calculate the *t*-statistics-based measurements to assess the cell type-specificity of genes within each tissue. (iii) We constructed a comprehensive association map of cell types and the human complex traits and diseases through conducting the cell type-specific enrichment analysis (CSEA) for thousands of traits we curated and maintained. (iv) We identified the trait-associated cell types, which will be good candidates to allocate the ‘causal’ or relevant cell types and shed light on the underlying mechanisms. (v) We highlighted those traits associated with the same cell type, which indicates the potential comorbidity and shared genetic basis (such as genes and pathways). (vi) All the curated data and associations have been managed and displayed in a user-friendly database to serve as the public repository of an omnibus map for human complex trait and cell type associations. (vii) Finally, we constructed a gene expression portal at cellular level to allow users to query and compare the relative abundance for genes of interest cross human tissues and cell types.

## DATA COLLECTION, ANALYSIS AND APPLICATION

### GWAS summary statistics collection and update

We adapted the ∼5000 GWAS summary statistics collected from the previous TSEA-DB (Tissue-Specific Enrichment Analysis DataBase) frozen by 19 June 2019. We further curated 260 GWAS traits from GWAS Catalog (15) and GRASP (grasp.nhlbi.nih.gov/) updated until 18 June 2020. Briefly, We collected the GWAS summary statistics from three major collections: the multi-trait collection (MTC) panel, which is a fixed collection of curations by previous studies (16–18); the UK Biobank (UKBB) panel, which deposited the UKBiobank ‘GWAS round 2’ results preprocessed by Neale's lab (http://www.nealelab.is/uk-biobank) on 1 August 2018 as the largest and most comprehensive resource of UKBB GWAS; the expanded trait collection (ETC), which is under recurrent curation from the new GWAS collected from GWAS Catalog and other resources. Both MTC and ETC panels were defined as the non-UKBB panel in our database.

### Quality control of GWAS data

We adapted the same quality control (QC) strategies for the updated GWAS summary statistics in the ETC panel. Briefly, we only used GWAS conducted in European ancestry for this database. No trans-ethnic meta-analysis GWAS was included since no proper linkage disequilibrium information could be applied to them. We further filtered those GWAS with lambda <0.8 or >1.3 to exclude deflated and inflated studies.

### Calculation of gene-based *P*-value and traits-associated-gene set

We updated our pipeline and applied a commonly used tool, Multi-marker Analysis of GenoMic Annotation (MAGMA v1.07) (19), to calculate the gene-level *P*-value. Specifically, we considered all SNPs in the gene body and 50 kb upstream and 35 kb downstream regions. We used the mean χ^2^ statistic for these SNPs to obtain gene-based *P*-values, considering the effects of the gene length, SNP density, and local linkage disequilibrium (LD) structure. We used the 1000 Genome Project Phase 3 European population as the reference panel.

We further used a dynamic threshold for trait-associated-gene (TAG) sets given the different significance from each GWAS study. The gene-based *P*-value generated by MAGMA was stratified to groups by threshold *P* < 0.05, *P* < 0.01, *P* < 0.001, *P* < 1 × 10^−4^ and *P* < 1 × 10^−5^. We further limited the number of genes in each group into the range from 20 to 3000, aiming to avoid statistical significance biased by genes set size in TAG. For each GWAS study, we required at least one TAG set complying with the criteria for the number of genes. Overall, we obtained 432 in the MTC panel, 316 in ETC panel, and 4372 in the UKBB panel stored in our CSEA-DB (Table 1).

**Table 1.** Summary of GWAS panel curation

Panel	Summary statistics	Number of TAG sets^a^
MTC^a^	432	1235
ETC^a^	316	1169
UKBB^a^	4372	11 370
**Total**	**5120**	**13 774**

^a^MTC: multi-trait collection panel, ETC: expanded trait collection panel, UKBB: UK Biobank collection panel, TAG: traits-associated-gene.

This table describes the distribution of 5120 GWAS summary statistics in three collection panels. The qualified GWAS traits-associated-gene (TAG) sets in each panel are listed in the third column.

## DATA COLLECTION

### Human organ system tissue single-cell transcriptome data

We conducted a deep literature-mining for human tissue- single-cell transcriptome data. We downloaded the datasets from three major sources, Human Cell Landscape (http://bis.zju.edu.cn/HCL/) (14), Single Cell Expression Atlas (https://www.ebi.ac.uk/gxa/sc/home), and extensive literature curation (9–13,20–22) (Supplementary file S1). We only collected the healthy tissue single-cell transcriptome data with detailed cell type annotation by original works. Firstly, we collected and curated the transcriptome matrix by tissue. We excluded those genes expressed in <30 cells in each tissue. Considering the statistical power, we further filtered those cell types with the number of cells no smaller than 30 in each tissue. Overall, we curated 71 tissue samples (55 unique tissues) and 752 tissue cell types (TCs) in adult and fetal tissues. The total number of genes in different tissues range from 3427 in adult ascending colon to 21 758 in placenta decidua.

The cell numbers of each cell type in each study were also recorded in the database as the information for cell type distribution in each tissue. The total number of cells in each tissue panel range from 995 in pancreas to 94 257 in spleen (12,20). We also provided the resource information and download link for each study for users to download (Supplementary file S1)

### Tissue-Cell type (TC) structure

We used a hierarchical structure to store the cell type in transcriptome data. Under the assumption that the cell types work collaboratively within each tissue, their tissue context should serve as another layer of information. We generated a unique tissue-cell type (TC) id for each of the cell types identified from the focal tissue single-cell data. This structure is displayed on the front page and the Browse function.

## UNIFORM PROCESSING PIPELINE

We constructed a standardized pipeline to preprocess the single-cell transcriptome datasets from different resources.

### Read count normalization

Read count matrix was obtained from each single-cell data resource. Pre-normalized data were also reversed to the read count matrix. Then, the matrix was normalized to counts per millions mapped reads. *G*_*i*_ is gene read count or Unique Molecular Identifier (UMI); *N_i_* is the total mapped read count in each cell; 10^6^ is the scale factor. The CPM matrix was further added by 1 and subsequently logistic transformed by 2 to eliminate the effect of extreme values.

$$\begin{equation*}{\rm CPM}_{\rm{i}} = {\rm{\;}}\frac{{{{\rm{G}}_{\rm{i}}}}}{{\frac{{{N_{\rm{i}}}}}{{{{10}^6}}}}}\end{equation*}$$

## TISSUE AND CELL TYPE ONTOLOGY ANNOTATION

### Tissue anatomy ontology

**Figure 1.**
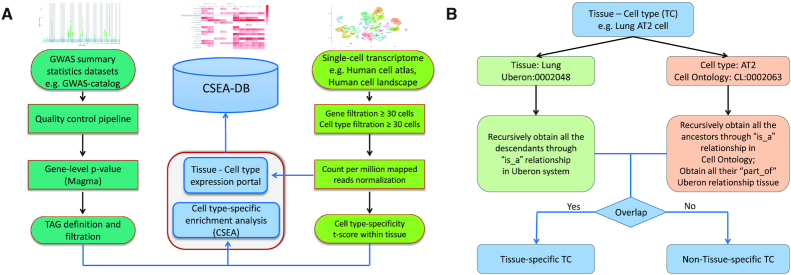
Workflow for Cell type-Specific Enrichment Analysis DataBase (CSEA-DB). (**A**) Workflow for CSEA-DB and statistics; Three figures (Manhattan plot, heatmap, and t-SNE plot) illustrate the features of the GWAS summary statistics dataset, Cell Type-Specific Enrichment Analysis Database (CSEA-DB), and single-cell transcriptome dataset, respectively. We process the datasets and construct the database based on this workflow. (**B**) Workflow for annotating tissue-cell types and identifying its tissue-specificity. For each tissue-cell type maintained in our database, we manually curate its ontology information in Uberon system and Cell Ontology and identify its tissue-specificity through this workflow.

We collected 55 unique human tissues from 11 distinct organ systems of the human body according to the anatomy. We further annotated them with their id in the Uberon system (23), which is an integrated cross-species anatomical ontology system. For each study, we recorded two Uberon ids (author-inferred tissue id and CSEA-DB annotated tissue id) for each tissue. If the study provides the tissue Uberon id, we kept the information as the author-inferred tissue and set as NA for tissue without author annotation. For CSEA-DB annotated tissue, we annotated its highest level Uberon system node. This CSEA-DB annotated tissue Uberon id would be used in the later on cell type annotation to determine whether the cell type is tissue-specific. The Uberon ontology obo file was downloaded from http://purl.obolibrary.org/obo/uberon.obo (accessed on 6 July 2020). We used the ‘is_a’ relationship to obtain all the descendants for each of the CSEA-DB annotated tissue id as the ‘tissue Uberon id set’ (Figure 1B).

### Cell type ontology

Another important feature of our CSEA-DB is all the cell type information adapted from the single-cell transcriptome data were further curated by CSEA-DB according to the ‘inferred cell type name’ from the original study. Specifically, we manually queried the ‘tissue name’ + ‘inferred cell type name’ in Cell Ontology (https://www.ebi.ac.uk/ols/ontologies/cl) (24). We recorded the most relevant cell type name and id returned from the Cell Ontology. Thus, each unique tissue cell type stored in our database have ‘inferred cell type name’, ‘Cell Ontology id’, and ‘Cell Ontology full name’. All the queries at the Cell Ontology website were conducted by 27 July 2020. We also downloaded the Cell Ontology obo file from https://github.com/obophenotype/cell-ontology (accessed on July 6, 2020). This file includes all the Cell Ontology id information and some annotated with Uberon id information through ‘part_of’ relationship. Firstly, we obtained all the Cell Ontology ancestors id through recursive ‘is_a’ relationship as a union for one focal cell type. Next, we searched all the possible ‘part_of’ relationships with annotation of the Uberon id (Figure 1B). Thus, we obtained a ‘cell type Uberon id set’ for all the focal Cell Ontology id in our database.

### Definition of tissue-specific cell types

Due to the wide-spreading connective tissue cell, including fixed cells (fibrocytes and adipocytes) and ‘wandering cells’ such as leukocytes (25,26), they might not have the cell type-specificity or directly related to the tissue function. Thus, we utilized an ontology-based method to check whether these tissue-cell types (TCs) were annotated as one cell type belongs to the corresponding tissue ontology. Specifically, for each of our 752 TCs, we overlap its Uberon id set described in the previous ‘**Cell type ontology**’ session with its ‘tissue Uberon id set’. If there is an overlapping of these two Uberon id sets, we would annotate this focal TC to tissue-specific or non-tissue-specific cell type, respectively (Figure 1B).

### Application of cell type-specific enrichment analysis

We modified our previously developed ‘tissue-specific enrichment analysis’ (5) and applied it to explore the cell type-specificity within each tissue. Briefly, we used the log_2_ (CPM + 1) normalized single-cell transcriptome matrix to calculate the cell type-specific expression within the cell types (number of cells ≥ 30) in each tissue. The *t*-statistics (*t_ij_*) for the coefficient of lm(*y_i_*∼*x_j_*) is calculated, where *y_i_* is a vector of the normalized expression of *i* gene; *x_j_* is a design matrix indicating the cells either in or outside of the *j* cell type; lm is the linear model regression. Thus, *t_ij_* represents the *t*-statistics of i gene in *j* cell type. Then we defined the top 5% *t*-statistic score gene in focal cell type as the cell type-specific genes. Lastly, we conducted a fisher exact test whether the TAG set from each trait is overrepresented with the cell type-specific genes, where *P*-value indicates the significance of this CSEA.

## DESCRIPTION OF THE WEBSITE AND TOOLS

### Overview of CSEA-DB

The front page of CSEA-DB includes an overview of human 11 organ systems. We used a hierarchical structure to store the tissue single-cell data information. The navigation bar has four featured functions ‘Browse’, ‘Search’, ‘Multi-trait’ comparison, and ‘scExpression’, Users can navigate the whole database through these four featured functions. The ‘Browse’ function stores all the trait and cell type maps split by trait panels (MTC, ETC and UKBB) as well as the trait-associated TCs map and TC-associated trait map. The search function supports the fuzzy search for trait name, tissue, and cell type of interest. And the return page includes all the possible results related to the keywords.

### Trait-associated TCs page

**Figure 2.**
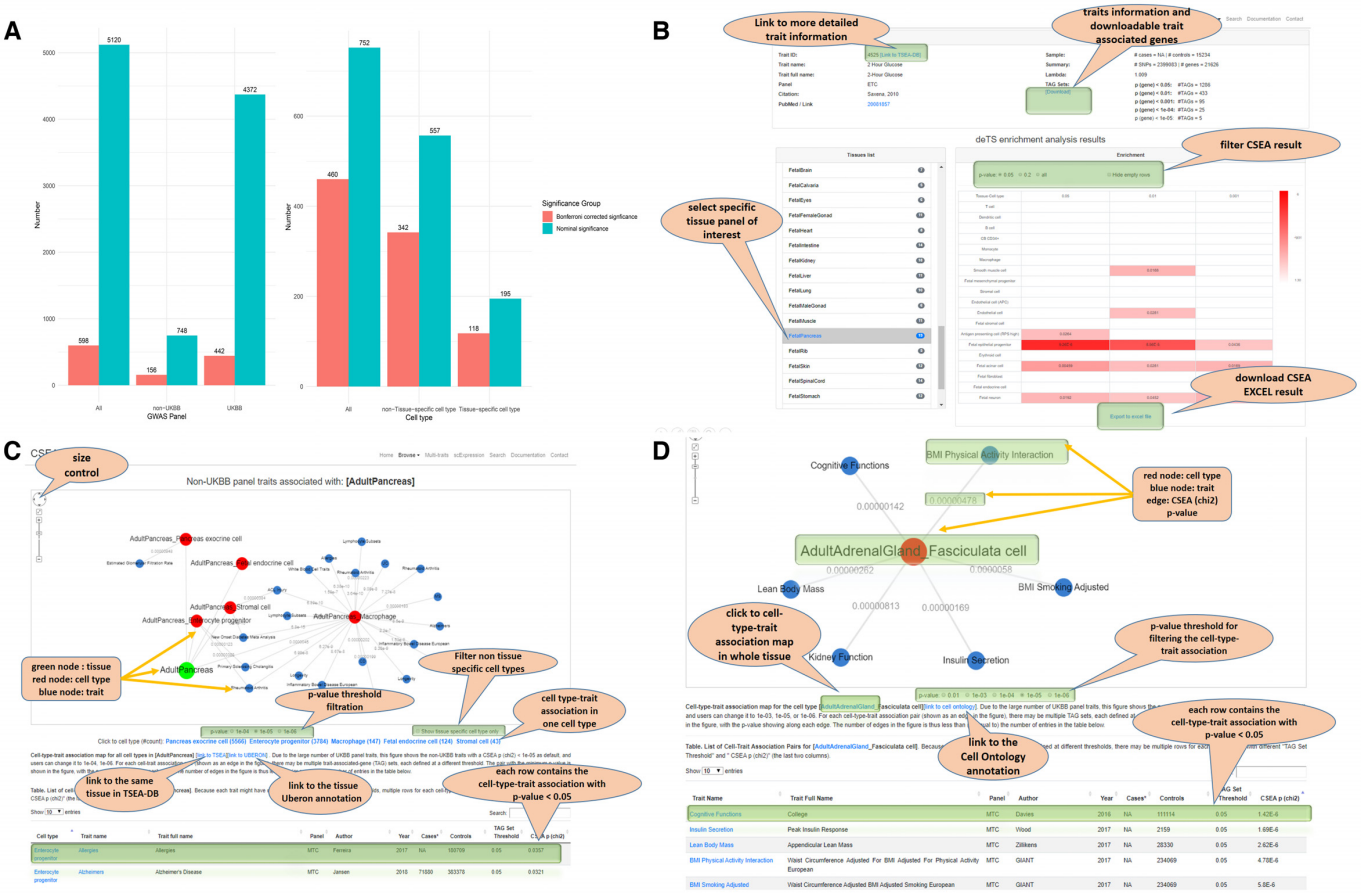
Statistics of trait and tissue-cell type association map and vignettes for database functions. (**A**) Trait and tissue-cell type map statistics; left: number of trait and tissue-cell type associations with at least one nominal or Bonferroni corrected significant association in at least one of the 752 tissue-cell types (TCs); y-axis is the number of unique traits; x-axis is the three categories, all GWAS traits, UK Biobank (UKBB) panel traits, and non-UKBB panel traits; right: number of trait and tissue-cell type associations with at least one nominal or Bonferroni corrected significant association in at least one of 5,120 GWAS traits; y-axis is the number of unique TCs; x-axis is three categories, all TCs, non-tissue-specific TCs, and tissue-specific TCs. (**B**) Cell type-specific enrichment analysis (CSEA) result from browsing the GWAS trait page. Top rows show trait information, including trait name, links to related sources, study summary, and trait-associated-gene (TAG) sets used for CSEA. The heatmap table in the bottom shows the enriched results of the trait in different cell types. Users can check the results in different tissues. The heatmap table is interactive and allows users to set different *P*-value thresholds for displaying the enriched results. The empty row which means there is no enriched result at the selected *P*-value level can be hidden by checking the option box. If the user selects the option ‘all’, all the enriched result values will be displayed in the heatmap table, only values that are lower than 0.2 will be marked with color proportions. (**C**) Network view of tissue-cell types-associated traits. Green node denotes a tissue, red nodes are cell types in this focal tissue, and blue nodes are associated traits with the cell types. The edges are labeled with *P*-values. The network view can be modified by setting different *P*-value cutoffs or hide non-specific cell types. A short note shows the description of the network data source. At the bottom, the data table shows a full list of cell type-trait association pairs for the tissue. Because each trait might have multiple TAG sets defined at different thresholds, multiple rows for each cell type-trait pair with different ‘TAG Set Threshold’ and ‘CSEA p (chi2)’ (the last two columns). (**D**) Network view of one specific cell type associated traits. This page is similar to the page in (C).

In the current CSEA-DB, we conducted the CSEA for 13 774 TAG sets (size ≥20 and ≤ 3000) from 5120 GWAS data sets over 752 TCs (Table 1). We found 99.97% of TAG sets (13 770 /13 774) have at least one cell type associated with a nominal *P*-value <0.05. After Bonferroni correction for 13 774 TAG sets and 752 TCs (∼5 × 10^−9^), we still observed 9.92% of TAG sets (1367/13 774) having at least one cell type. At the GWAS traits level, we observed 100% (5120/5120) and 11.68% (598 /5120) of GWAS have at least one cell type with significant associations before and after multiple-testing correction. Specifically for the non-UKBB panel, we observed 100% (748 /748) and 20.86% (156 /748) of GWAS identified with the significant association before and after multiple-testing correction (Figure 2A, left). Thus, we identified that non-UKBB GWAS traits tend to have a higher proportion of Bonferroni corrected significance than the UKBB GWAS traits. Figure 2B shows an overview for one specific GWAS trait and its basic information, including trait name, case and control number, reference, and TAG sets information. The CSEA results for the trait could be displayed in all TCs or one tissue and its corresponding TCs.

### TC-associated traits page

Among 752 TC, 100% TCs has at least one nominal significant association with one GWAS trait, while 61.17% TCs (460 /752) have at least one Bonferroni corrected significance with at least one GWAS phenotype (Figure 2A, right). We did not observe the tissue-specific cell types (60.51%, 118/195) have a different proportion of Bonferroni corrected significance (comparing to nominal significance) from the non-tissue-specific cell types (61.40%, 342/557). We aimed to explore the TC-associated traits both tissue-wisely and TC-wisely. As shown in Figure 2C, the TCs and traits are connected by edges with *P*-value of association smaller than a certain threshold, while TCs are connected with their corresponding tissue. Users can filter the *P*-value threshold if the network is too sparse or too dense. Below the threshold, we also provide the information of cell types and their numbers within this tissue. Users can click the hyperlink to look into the specific TC-associated traits. Interestingly, we identified some of the cell types from the same tissue that might share strong associations with the same trait, indicating that these cell types might work together to contribute to the corresponding GWAS phenotype. As shown in Figure 2D, one specific TC-associated traits page only contains the association of one TC. This page is similar to the association page for TCs or traits as shown in Figure 2C.

### Bridging to TSEA-DB

In addition to our CSEA result, we provide a hyperlink to TSEA-DB for each trait to allow users to browse the traits-associated tissues (TATs). TSEA-DB provides a comprehensive overview of each trait by displaying the Manhattan plot for GWAS summary statistics and gene-level *P*-values. Moreover, users could compare both results of TSEA and CSEA in shared tissues, providing deeper biological insight of genetic signals at cellular resolution as well as rescuing those diluted signals in the bulk tissue expression.

### scExpression page

The scExpression page provides normalized gene expression information at both tissue and cell type level. Users can submit one gene symbol name of interest and the scExpression page will return an overview (barplot) of the average CPM normalized gene expression within each of the 68 tissues with UMI-based data. The three SMART-seq2 datasets based on the full-length RNA-seq method are not listed, as their normalized expression profiles are not comparable with other UMI-based data (27,28). Once the users further click the tissue bar of interest, it will generate barplot for the average CPM normalized expression of each cell type in that tissue. This page is a useful tool for researchers who aim to check the gene expression across human tissues and cell types. Overall, we provide a one-stationary curation for the average normalized gene expression of tissue and cell type.

### Multi-trait comparison function

As we demonstrated in the trait-associated cell type, containing >5120 traits information and their CSEA result across over 752 TCs tissue-wisely or together. Since there are multiple studies for the same or similar trait, the comparison of these traits could help to identify the consistent enriched cell types shared by the same trait from different studies. Moreover, multiple tissues or TCs might be related to complex traits and diseases. Thus, we provide this multi-trait comparison function to explore the shared or unique cell types across multiple traits and multiple TCs of interest.

**Figure 3.**
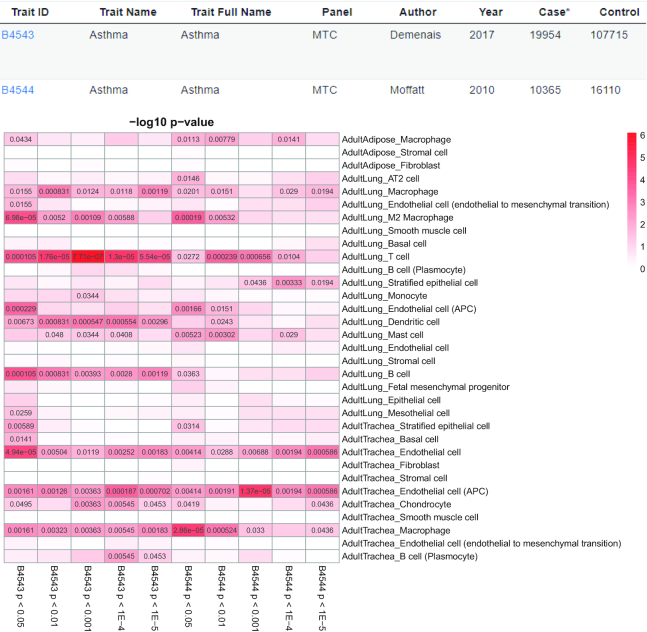
Multi-trait multi-cell types comparison using two asthma studies in three human tissues (Adult Adipose, Adult Lung and Adult Trachea). An example of our Multi-trait function by comparing the asthma GWAS from two datasets in three tissues (33 tissue-cell types). Our ‘Multi-trait’ function allows users to compare at most 10 data sets in all the tissue-types in our database at one time. The top two rows demonstrate the study information about these traits and the heatmap below is the cell type-specific enrichment analysis (CSEA) for the GWAS with multiple trait-associated-gene (TAG) sets. The color is proportional to the –log(*P*) value of the associations. The cells with *P* < 0.05 are filled with the corresponding *P*-values and the tables with *P* ≥ 0.05 are left blank.

To this end, this multi-trait comparison function could explore the associations between multiple traits and multiple TCs of interests simultaneously. We used ‘Ashma’ as an example in Figure 3. Asthma is a condition that leads to the inflammation in the airways and the bronchial tubes that carry air into the lung, which makes patients difficult to breathe (29). We selected two Asthma studies with 10 TAG sets and all TCs in three tissues, including two disease-relevant tissue trachea and lung, and one disease irrelevant tissue adipose through the multi-trait comparison function.

We identified the same cell types might act differently in disease-relevant and irrelevant tissues. For instance, we found macrophage has most significant association with *P* > 0.005 in adipose, *P* = 6.98 × 10^−5^ in lung, and *P* = 2.86 × 10^−5^ in trachea. We also identified endothelial cell (APC) has relatively high risk in both lung (*P* = 2.29 × 10^−4^) and trachea (*P* = 1.37 × 10^−5^), although T cell in lung has the most significant association (*P* = 7.71 × 10^−7^). Nevertheless, we also find the lung B cell has the association of *P* = 1.05 × 10^−4^, while the plasma B cell has no association with *P* < 0.05 in any of 10 TAG sets. Overall, this systematic characterization of the TCs underlying genetic signals could help to dissect the potential mechanism of asthma.

### TSEA and CSEA comparison

**Figure 4.**
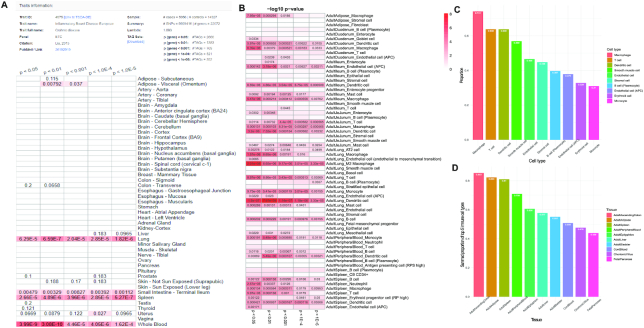
Comparison between TSEA-DB and CSEA-DB for Crohn's disease, and cell type distribution among tissues in CSEA-DB. (**A**) Tissue-specific enrichment analysis (TSEA) results for one Crohn's disease GWAS across 47 tissues in Genotype-Tissue Expression (GTEx). Top rows are the basic information about the Crohn's disease GWAS, the heatmap below demonstrates the TSEA results of this Crohn's disease GWAS with multiple trait-associated-gene (TAG) sets from the Tissue-Specific Enrichment Analysis DataBase (TSEA-DB). (**B**) Cell type-specific enrichment analysis (CSEA) results of Crohn's disease GWAS in tissue-cell type level with multiple TAG sets from CSEA-DB. (**C**) Barplot for top 10 frequently identified cell types in CSEA-DB curated tissue transcriptome datasets.). (**D**) Barplot for top 10 tissues with the largest summed proportions of the five lymphatic cells (macrophage, T cell, dendritic cell, B cell (plasmocyte), and monocyte).

To understand the relationship between TSEA and CSEA, we compare their results for one autoimmune disease (Crohn's Disease) in TSEA-DB and CSEA-DB (Figure 4A and B) (4). Specifically, we identified five associated tissues (adipose visceral, lung, small intestine, whole blood, and spleen) that have at least one TAG with *P* < 0.01 in the TSEA-DB. (Figure 4A). The top associated tissues are whole blood (*P* = 3.08 × 10^−10^), spleen (*P* = 5.27 × 10^−7^), lung (*P* = 6.59 × 10^−7^), small intestine (*P* = 0.001), and adipose visceral (*P* = 0.008). We selected the CSEA result from the corresponding tissues (AdultAdipose, AdultDuodenum, AdultIleum, AdultJeJunum, AdultLung, AdultPeripheralBlood and AdultSpleen) in CSEA-DB (Figure 4B). Since the cell type-specificity is calculated within each tissue, their significance is comparable within each focal tissue. We identified that dendritic cell (7.81 × 10^−9^), M2 macrophage (2.01 × 10^−8^) and M2 macrophage (1.57 × 10^−6^) are the top significant cell types in lung. Interestingly, we found dendritic cell (3.20 × 10^−6^), macrophage (5.47 × 10^−6^), and endothelial cell (APC) (3.18 × 10^−6^) are the most enriched cell types in three small intestine tissues (disease-relevant tissues). Moreover, endothelial cells (APC) in non-small intestine tissues all have *P* > 0.001, suggesting that the endothelial cell (APC) might work differently in small intestine tissues and contribute to the Crohn's disease along with the dendritic cell, and macrophage. Besides, the proportions of ‘causal’ cell types within each tissue could also be an important indicator to assess their effect. Lastly, the genetic factors could only explain a small proportion of the disease. The microenvironment that tissues are exposed to might have contributions to the disease pathogenesis.

### Documentation page

We built a documentation page to briefly describe the data collection, preprocessing, and analysis (https://bioinfo.uth.edu/CSEADB/document.php). We also provide vignettes for website functions, including Browse, Search, Multi-trait comparison and scExpression functions.

## DATABASE DESIGN AND UPDATES

The CSEA-DB web interfaces were constructed with standard HTML and Bootstrap 4 libraries (http://getbootstrap.com/). The data were processed using R and python scripts. The processed and annotated data and summary statistics were stored in MySQL. PHP was the main language used for implementing the functions of CSEA-DB, such as, browsing, searching and data exporting. The interactive and dynamic web pages were implemented through several JavaScript libraries (CytoscapJS, zTReeJS, HighchartJS) and Ajax strategies. Our database could be easily expanded with the newly updated data through highly efficient scripts. In the previous work TSEA-DB (7), we have built a standardized workflow to select, preprocess, and conduct quality control on the GWAS summary statistics. In this work, we used that pipeline and updated 171 qualified GWAS summary statistics since the last update on 19 June 2019. We will update GWAS summary statistics annually. In this work, we built a pipeline to process the single-cell tissue transcriptome data, including data quality control, ontology annotation and cell type-specific expression panel construction. Due to rapid advances in single-cell genomics technology, we will seasonally update our single-cell dataset collection to include the newly available human tissues and related cell types. The CSEA could be implemented using modified scripts based on our deTS package (5). All the results in this work are based on the release on 13 August 2020.

## CONCLUDING REMARKS AND FUTURE DEVELOPMENT

In previous TSEA-DB (https://bioinfo.uth.edu/TSEADB/), we have successfully decoded the diseases relevant tissues, most of which aligned with the current knowledge. In this Cell type-Specific Enrichment Analysis DataBase (CSEA-DB, https://bioinfo.uth.edu/CSEADB/), we mine the various data deeper into the trait-associated tissue cell types. We updated and reprocessed the long-termly maintained GWAS summary statistics with the new pipeline. We collected, annotated, and processed 71 single-cell transcriptome studies covering 55 unique adult and fetal tissues in 11 human organ systems.

Interestingly, we observed a broad expression of immune cells among human tissues. As shown in Figure 4C, we found human macrophage, T cell, dendritic cell existed in 74.1%, 63.8%, 63.8% out of 58 tissue from HCL, respectively. This discovery supports the widespread connective tissue cells such as macrophage, T cell, and dendritic cell in human tissues. Moreover, we summed up the cellular proportion of top five lymphatic cell types within each human tissues and identified that ascending colon (85.3%), adipose (82.3%), spleen (81.0%), peripheral blood (70.9%), and epityphlon (appendix) (60.6%) are the top five human tissues that contain the largest proportion of these five lymphatic cells (macrophage, T cell, dendritic cell, B cell (plasmocyte) and monocyte) (Figure 4D). Considering the wide-spreading lymphatic cells in human tissues, we did a systematic curation for cell types identified in the transcriptome data with those connective cells in each tissue. However, the ‘tissue-specific’ and ‘non-tissue-specific’ cell types are not a rigorous biological definition. We provided this cell type filtration option to eliminate the potential ‘noise’ from the non-tissue-specific connective cells such as fibrocytes, adipocytes, and leukocytes cells, highlighting the associations of ‘tissue-specific’ cell types. Overall, CSEA-DB could provide systematic potential insights into the biological mechanisms of human complex diseases at cellular resolution.

In the future, CSEA-DB aims to update the database in the following three directions. (i) Current CSEA-DB only curates the GWAS with samples of European Ancestry. With the recent advent of GWAS in other populations (e.g. African and East Asian population), we will integrate the more population panel into our GWAS curation as well as the annual update referring to GWAS-catalog. Moreover, the chromatin interaction information for chromosome 3D data has also been integrated to better interpret the effect of variants in long-term chromosomal interactions (30,31). We will actively update our pipeline to better assess the gene-level *P*-value from GWAS summary statistics. (ii) We will continuously collect and curate the emerging single-cell transcriptome data quarterly. We will have a more comprehensive collection of human single-cell transcriptome data along with the advance of ongoing projects like Human cell atlas and single-cell expression atlas with more data and more accurate annotation. Since more single-cell platforms adapted the UMI-based method (32), we expect to integrate more single-cell transcriptome datasets (e.g. developmental and temporal-spatial data) into our database. (iii) In this study, we observed that some specific complex human disease is significantly associated with multiple cell types in one specific tissue, suggesting these cell types from this tissue might all contribute to the etiology of diseases. As shown in Figure 3, we found that both immune cells and epithelial cells (AT2) are all extremely enriched in human adult lung, indicating their co-occurrence might be associated with the underlying mechanism. Nevertheless, different cells within certain microenvironments will communicate with each other and coordinate to transduce signals (such as immune response in immune cells) (33). Deciphering the genetic risks underlying such intercellular interactions will further help us to understand the etiology of human complex traits and diseases (34).

In summary, we constructed an omnibus map for over 5120 human GWAS phenotypes and 752 human tissue cell types. We identified many tissue-specific cell types that play crucial roles; and such results align with previous discoveries in TSEA-DB. Meanwhile, some widespread cell types might play different roles in different tissues and contribute to the disease pathogenesis contextually. Moreover, tissue cell type could be related to multiple GWAS phenotypes and multiple cell types carrying genetic risks within one tissue might communicate and work collaboratively. These discoveries could provide new insights into understanding the mechanism of human complex traits and diseases.

## Supplementary Material

gkaa1064_Supplemental_File
